# Ureteroscopy is more cost effective than shock wave lithotripsy for stone treatment: systematic review and meta-analysis

**DOI:** 10.1007/s00345-018-2320-9

**Published:** 2018-05-05

**Authors:** Robert M. Geraghty, Patrick Jones, Thomas R. W. Herrmann, Omar Aboumarzouk, Bhaskar K. Somani

**Affiliations:** 1 Department of Urology, University Hospital Southampton NHS Trust, Southampton, SO16 6YD UK; 2 Clinic of Urology, Spital Thurgau AG, Frauenfeld, Switzerland; 3 Department of Urology, Queen Elizabeth University Hospital, Glasgow, UK

**Keywords:** Ureteroscopy, Shock wave lithotripsy, Cost, Effectiveness, Outcomes

## Abstract

**Introduction:**

A rising incidence of kidney stone disease has led to an increase in ureteroscopy (URS) and shock wave lithotripsy (SWL). Our aim was to compare the cost of URS and SWL for treatment of stones.

**Methods:**

A systematic review and meta-analysis based on Cochrane and PRISMA standards was conducted for all studies reporting on comparative cost of treatment between URS and SWL. The cost calculation was based on factual data presented in the individual studies as reported by the authors. English language articles from January 2001 to December 2017 using Medline, PubMed, EMBASE, CINAHL, Cochrane library and Google Scholar were selected. Our study was registered with PROSPERO (International prospective register of systematic reviews)—registration number CRD 42017080350.

**Results:**

A total of 12 studies involving 2012 patients (SWL-1243, URS-769) were included after initial identification and screening of 725 studies with further assessment of 27 papers. The mean stone size was 10 and 11 mm for SWL and URS, respectively, with stone location in the proximal ureter (*n* = 8 studies), distal ureter (*n* = 1), all locations in the ureter (*n* = 1) and in the kidney (*n* = 2). Stone free rates (84 vs. 60%) were favourable for URS compared to SWL (*p* < 0.001). Complication rates (23 vs. 30%) were non-significantly in favor of SWL (*p* = 0.11) whereas re-treatment rates (11 vs. 27%) were non-significantly in favor of URS (*p* = 0.29). Mean overall cost was significantly lower for URS ($2801) compared to SWL ($3627) (*p* = 0.03). The included studies had high risk of bias overall. On sub-analysis, URS was significantly cost-effective for both stones  < 10 and  ≥ 10 mm and for proximal ureteric stones.

**Conclusion:**

There is limited evidence to suggest that URS is less expensive than SWL. However, due to lack of standardization, studies seem to be contradictory and further randomized studies are needed to address this issue.

## Introduction

The worldwide incidence of kidney stone disease (KSD) is rising [1]. The use of ureteroscopy (URS) for KSD has also risen, whilst shockwave lithotripsy (SWL) use has fallen [2]. This trend has resulted from improvement of technique, minimization of scopes and better laser fragmentation technology [3]. While stones in the ureter and most stones up to 2 cm in the kidney are suitable for both URS and SWL, several prospective randomized controlled trials have demonstrated the superiority of URS over SWL in terms of stone free rate (SFR) and retreatment rates [4].

Majority of stones might be amenable for either URS or SWL and although treatment is tailored after patient counseling, for patients suitable for both modalities, a major factor in treatment selection is the cost associated with it, especially with healthcare resources already stretched to its limit. These costs can vary greatly depending on the initial purchase price, cost of consumables and repair, durability of the instruments, the negotiated discounts available from manufacturers and the reimbursement received by the providers.

While cost is increasingly an important factor in the decision-making, to date, there has been no review comparing the cost of URS and SWL. Although individual cost of URS and SWL has been mentioned, there is no clear way to compare costs due to discrepancies across various healthcare systems and partly because indirect costs are difficult to measure [5–8]. In the absence of clear cost comparison, we wanted to look the cost of surgical stone management as has been reported by the authors in studies comparing both URS and SWL. To this end, we perform a systematic review and meta-analysis of all studies reporting on comparative cost of treatment between URS and SWL.

## Methods and Materials

### Evidence acquisition: criteria for considering studies for this review

Population: Adults with ureteral or renal urolithiasis
Intervention: Ureteroscopy
Comparator: Shockwave lithotripsy
Outcome: Cost
Study design: Systematic review and meta-analysis

Inclusion criteria:

1. all published articles written in the English language
2. studies reporting on comparative cost of treatment between URS and SWL
3. URS will include rigid, semi-rigid and flexible.

Exclusion criteria:

1. studies examining treatment for non-urolithiasis conditions
2. older studies using the same data as a more recent study—the longest cohort was chosen to avoid duplication
3. grey literature and decision analysis models which did not have real patient data

### Search strategy and study selection

The systematic review was performed according to the Cochrane review guidelines [9]. The search strategy was conducted to find relevant studies from Ovid medline without revisions (2001–2017), PubMed (2001–2017), EMBASE (2001–2017), Cochrane Library (2017), CINAHL (2001–2017), Clinicaltrials.gov, Google Scholar and individual urologic journals.

The search terms used included: ‘ureteroscopy, ‘URS, ‘ureterorenoscopy, ‘retrograde intrarenal surgery’, ‘RIRS’, ‘shockwave lithotripsy’, ‘SWL’, ‘ESWL’, ‘cost’, ‘calculi*’, ‘stone*’, ‘nephrolithiasis’ and ‘urolithiasis’. Boolean operators (AND, OR) were used to refine the search.

As the cost data prior to 2001 was not relevant anymore, the search was limited to English language articles published between January 2001 and December 2017. Authors of the included studies were contacted in the case of data not being available or clear. If the authors did not reply data was estimated from the graphs and other data provided in the study and if the data could not be estimated, then the study was excluded from analysis. Our study was registered with PROSPERO (International prospective register of systematic reviews)—registration number—CRD 42017080350.

**Fig. 1:**
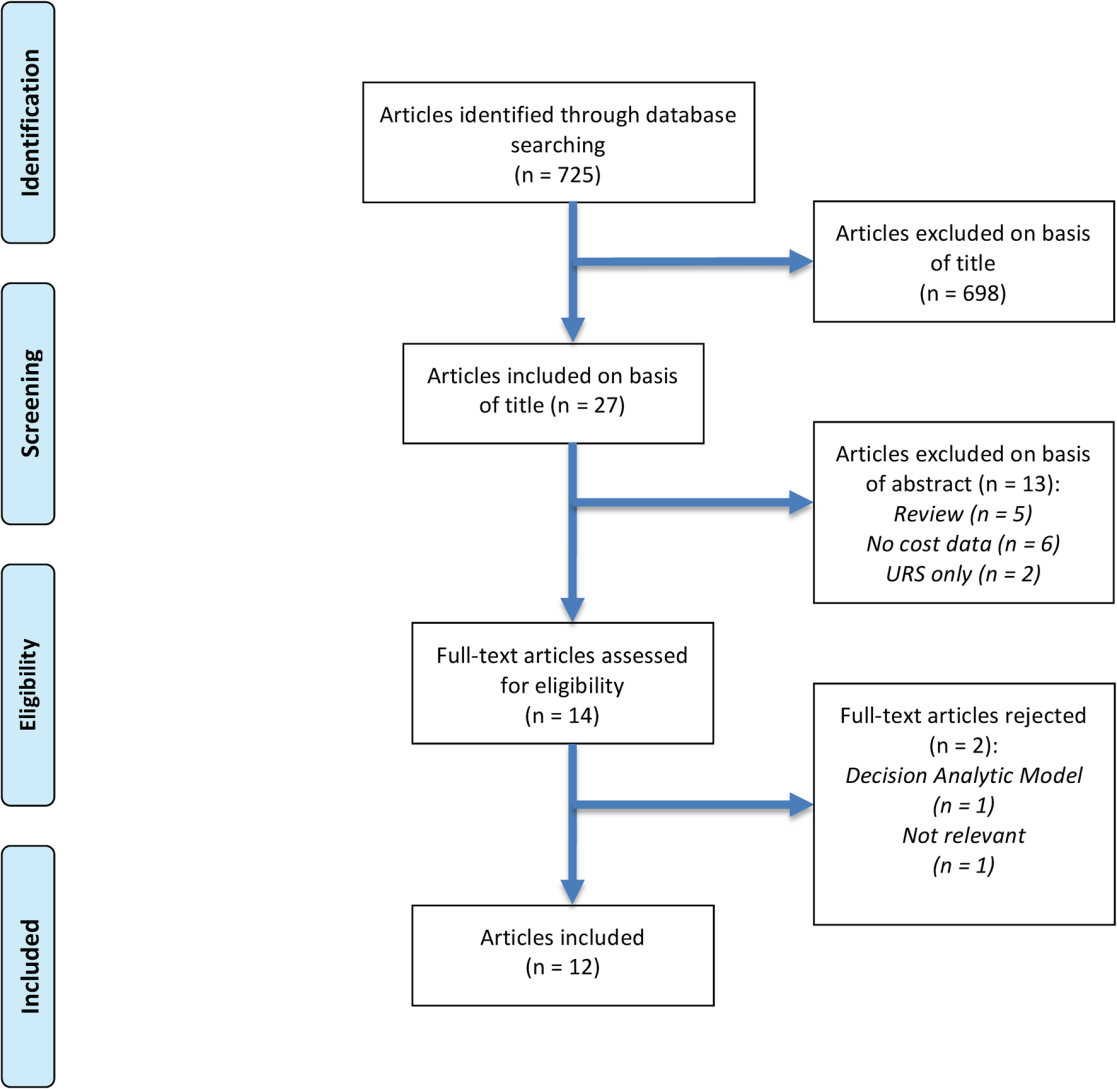
PRISMA flowchart of the included studies

Two experienced reviewers (RG and BS) identified all studies. All studies that appeared to fit the inclusion criteria were included for full review. Each reviewer independently selected studies for inclusion in the review and all discrepancies were resolved with mutual agreement and consensus with the third author (PJ) (Fig. 1).

### Data extraction and analysis

The following variables were extracted from each study: year of publication, country of study, study period, treatment modality, price/cost, age, stone size, location of stone, stone free rate, complications, hospital stay, retreatment rate and operative time. Bias analysis was performed using the GRADE guidelines [10].

Cost was converted to US dollars based on the mean exchange rate of the year of publication. Cost was rounded to the nearest dollar. Data was collated using Microsoft Excel (version 12.2.4) and analysed using Revman (version 5.0) and SPSS (version 21). Those studies with no standard deviations for cost were not given weight in the forest plot analysis. Forest plots were generated in Revman 5.3.

Continuous data was presented as standard mean difference and for dichotomous data risk difference was used. Data heterogeneity was assessed using a Chi squared test. If there were no significant heterogeneity then random effects were used. If there was a significant result, this was adjusted for using standard mean difference and random effects on forest plot analysis.

## Results

### Study characteristics

There were 12 studies examining the cost of URS vs. SWL [11–22]. These took place in the USA [11, 12, 20, 21], China, [13–15, 19], Egypt [16], Taiwan [17] and the UK [18, 22]. Seven of the included studies were retrospective cohort studies [12–14, 18–21] two were prospective cohort studies [17, 22] and the remaining three [11, 15, 16]. The studies took place over a mean 2-year period. Overall there were 2012 patients, with 1243 undergoing SWL and 769 undergoing URS. Studies were subdivided further according to stone size (see Tables 1, 2).

**Table 1:** URS vs. SWL study demographics

Study	Country	Study type	Study period	Patients, *n*	SWL, *n*	URS, *n*	Type of URS
Pearle et al. 2001 [11]	USA	Prospective randomized trial	1995–2000	64	32	32	Semi-rigid
Parker et al. 2004 [12] < 10 mm	USA	Retrospective cohort	1997–2001	154	73	81	Flexi/semirigid -unclear
Parker et al. 2004 [12] ≥10 mm	USA	Retrospective cohort	1997–2001	66	38	28	Flexi/semirigid -unclear
Wu et al. 2004 [13]	China	Retrospective cohort	2002–2003	80	41	39	Semi-rigid
Wu et al. 2005 [14] < 10 mm	China	Retrospective cohort	2002–2003	113	68	45	Semi-rigid
Wu et al. 2005 [14] ≥10 mm	China	Retrospective cohort	2002–2003	107	51	56	Semi-rigid
Lee et al. 2006 [15]	China	Prospective randomized trial	2001–2003	42	22	20	Semi-rigid
Salem 2009 [16] <10 mm	Egypt	Prospective randomized trial	N/A	110	58	52	Semi-rigid
Salem 2009 [16] ≥10 mm	Egypt	Prospective randomized trial	N/A	90	42	48	Semi-rigid
Huang et al. 2009 [17] < 10 mm	Taiwan	Prospective cohort	1998–1999	241	201	40	Semi-rigid
Huang et al. 2009 [17] ≥10 mm	Taiwan	Prospective cohort	1998–1999	207	159	48	Semi-rigid
Koo et al. 2011 [18]	UK	Retrospective cohort	N/A	88	51	37	Flexible
Cui et al. 2014 [19]	China	Retrospective cohort	2010–2012	160	80	80	Rigid
Cone et al. 2014 [20]	USA	Retrospective cohort	2010–2011	158	78	80	Flexible
Cone et al. 2017 [21]	USA	Retrospective cohort	2010–2011	113	51	62	Flexible (*n* = 39), semirigid (*n* = 23)
Chan et al. 2017 [22]	UK	Prospective cohort	2008–2013	219	198	21	Flexible
Total				2012	1243	769	

*N/A* not available

**Table 2:** SWL vs. URS patient and stone demographics

Study	Age, year ± SD (range)		Stone size, mm ± SD (range)		
	SWL	URS	SWL	URS	Location
Pearle et al. 2001 [11]	41.2 ± 14.9	41.2 ± 12.8	7.4 ± 2.3	6.4 ± 2.7	Distal ureter
Parker et al. 2004 [12] < 10 mm	50 ± 17	44 ± 15	< 10	< 10	Proximal ureter
Parker et al. 2004 [12] ≥10 mm	55 ± 15	48 ± 16	> 10	> 10	Proximal ureter
Wu et al. 2004 [13]	51	51	12.8 ± 0.4	15.1 ± 0.5	Proximal ureter
Wu et al. 2005 [14] < 10 mm	47.5 ± 1.5	51.0 ± 2.0	6.9 ± 0.2	7.2 ± 0.2	Proximal Ureter
Wu et al. 2005 [14] ≥ 10 mm	51.5 ± 1.9	53.8 ± 1.5	12.1 ± 0.3	17.0 ± 0.7	Proximal Ureter
Lee et al. 2006 [15]	54.2 ± 16.7	48.5 ± 13.3	17.9 ± 3.9	18.5 ± 2.9	Proximal ureter
Salem, 2009 [16] < 10 mm	42.8 (37–60)	41.2 (36–60)	6.2 (5–9)	6.8 (6–9)	Proximal ureter
Salem 2009 [16] ≥ 10 mm	45.4 (37–55)	36.7 (20–48)	12.5 (11–20)	12.2 (12–20)	Proximal ureter
Huang et al. 2009 [17] < 10 mm	52.5 ± 16.1	49.5 ± 12.7	<10	<10	Proximal Ureter
Huang et al. 2009 [17] ≥ 10 mm	52.5 ± 16.1	49.5 ± 12.7	> 10	> 10	Proximal ureter
Koo et al. 2011 [18]	51.2 ± 14.9	56.6 ± 15.9	< 20	< 20	Ureteric (all locations)
Cui et al. 2014 [19]	40.6 ± 9.8	41.5 ± 10.5	9.8 ± 3.5	10.2 ± 4.3	Proximal ureter
Cone et al. 2014 [20]	54 ± 15	47 ± 11	7.0 ± 0.27	7.27 ± 0.27	Renal
Cone et al. 2017 [21]	53 ± 13	54 ± 16	7.64 ± 3.32	7.50 ± 2.22	Proximal ureter
Chan et al. 2017 [22]	54.1 ± 13.3	62.2 ± 15	12.4 ± 2.4	13.1 ± 3.7	Lower pole renal
Total	49.4	48.5	10.2	11.0	

### Patient and stone demographics

The mean age of patients in the SWL group was 49.4 years (range: 37–60), and the URS group was 48.5 years (range: 20–60). Stone size was similar between the two groups with a mean size of 10.2 mm (range: 6.2–20 mm) for SWL and 11 mm for URS (range: 6.4–20 mm) (Table 2). There were five studies examining stones smaller than 10 mm [11, 12, 14, 16, 20], two studies examining stones less than 15 mm [20, 21] and five studies examining stones 10 mm and larger [12, 14, 16, 17, 22].

Eight studies compared treatment of proximal ureteric stones only [12–17, 19, 21]. The others compared distal stones [11], ureteric stones of all locations [18] and renal stones [20, 22].

### Intra- and post-operative characteristics

The studies predominantly used semi-rigid URS. Six studies used semi-rigid URS, three used flexible URS, one study using rigid URS, one study used either flexible or semi-rigid URS and one study did not specify the type of URS (Table 1).

**Fig. 2:**
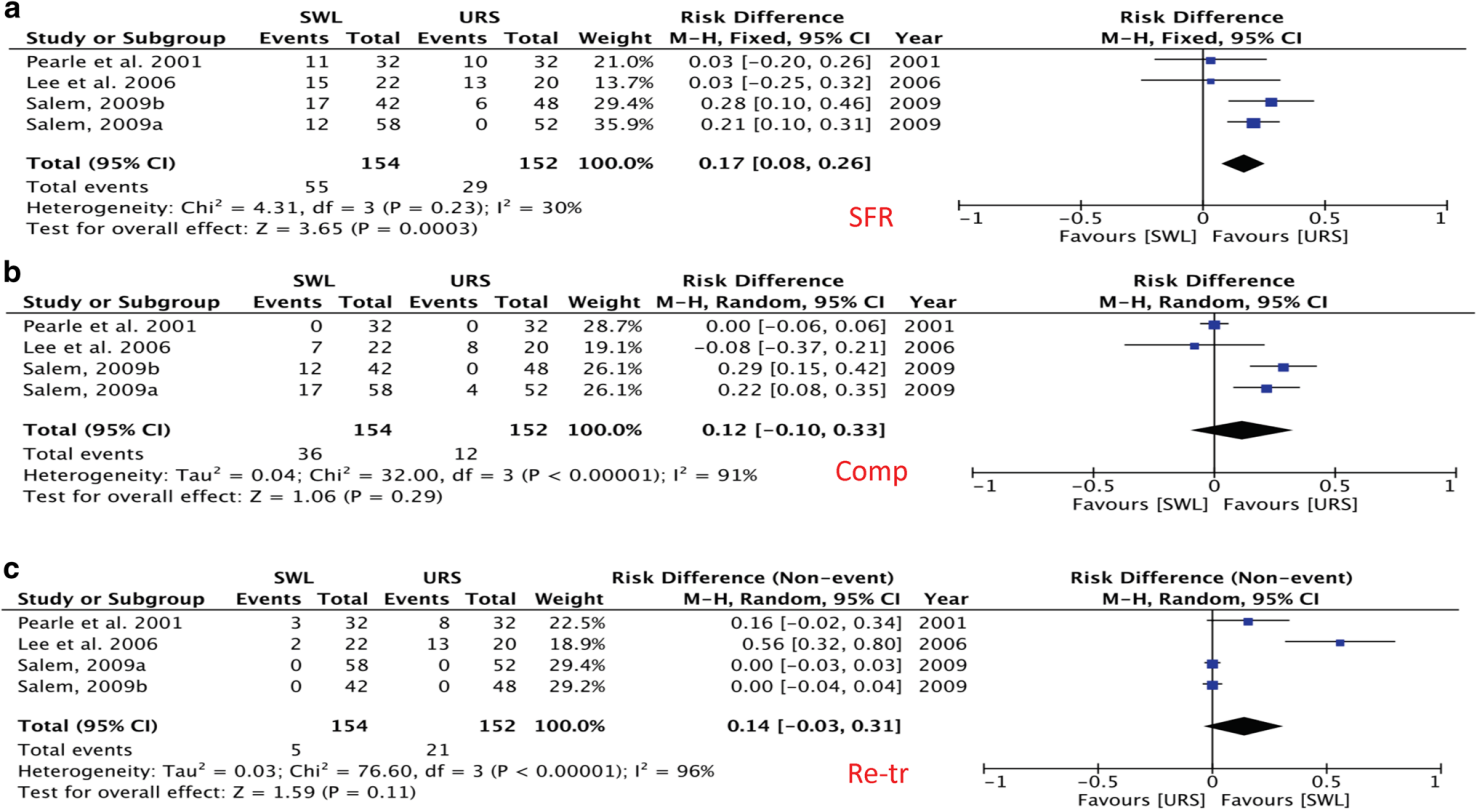
**a**–**c** forest plot of SFR, complications and re-treatment

The mean initial SFR was significantly higher for URS (84%) vs. SWL (60%). Comparison between the randomized trials demonstrated significantly higher stone free rates for URS (*I*^2^ = 30%: risk difference = 0.17, 95% CI 0.08–0.26, *p* < 0.001) (Table 3 and Fig. 2a).

**Table 3:** SWL vs. URS intra- and post-operative characteristics

Study	Initial SFR (%)		Complications, *n* (%)		Retreatment (%)	
	SWL	URS	SWL	URS	SWL	URS
Pearle et al. 2001 [11]	66%	69%	3 (9%)	8 (25%)	None	None
Parker et al. 2004 [12] < 10 mm	60%	90%	20 (27.4%)	19 (23.5%)	N/A	N/A
Parker et al. 2004 [12] ≥10 mm	45%	93%	17 (44.7%)	12 (42.9%)	N/A	N/A
Wu et al. 2004 [13]	61%	92%	None	None	39%	8%
Wu et al. 2005 [14] < 10 mm	85.30%	91.10%	N/A	N/A	14.7%	8.9%
Wu et al. 2005 [14] ≥10 mm	35.20%	76.80%	N/A	N/A	64.8%	23.2%
Lee et al. 2006 [15]	31.80%	35%	2 (9%)	13 (65%)	31.80%	40%
Salem, 2009 [16] < 10 mm	80%	100%	N/A	N/A	40.48%	8.33%
Salem 2009 [16] ≥ 10 mm	60%	88%	54 (93%)	27 (52%)	20.69%	None
Huang et al. 2009 [17] < 10 mm	75.60%	95.00%	N/A	N/A	24.4%	5%
Huang et al. 2009 [17] ≥ 10 mm	66.70%	85.40%	N/A	N/A	33.3%	14.6%
Koo et al. 2011 [18]	45.10%	64.90%	4 (8%)	4 (11%)	7.50%	2.50%
Cui et al. 2014 [19]	77.50%	97.50%	30 (38%)	31 (39%)	21.60%	16.20%
Cone et al. 2014 [20]	55%	95%	N/A	N/A	12.80%	5%
Cone et al. 2017 [21]	47.10%	88.70%	N/A	N/A	N/A	N/A
Chan et al. 2017 [22]	62.60%	76.20%	6 (3%)	3 (14%)	40%	10%
Total	60% ± 15%	84% ± 16%	136 (23%)	117 (30%)	27% ± 16%	11% ± 11%
*p* (χ^2^)	< 0.001		0.26		<0.001	
OR (95% CI)	4.58 (3.52–5.97)		0.72 (0.50–1.03)		3.43 (2.48–4.74)	
*p* (forest plot)	< 0.001		0.07		< 0.001	

*N/A* not available, *SWL* Shockwave lithotripsy, *URS* Ureteroscopy

The total number of complications for each group were 136 for SWL and 117 for URS. The mean complication rates were 23% for SWL and 30% for URS. There was no statistical difference between the two when examining the randomized trials (*I*^2^ = 96%, risk difference = 0.14, 95% CI − 0.03–0.31, *p* = 0.11) (Table 3 and Fig. 2b).

There were higher retreatment rates for SWL (27%) than for URS (11%). Meta-analysis of the randomized trials demonstrated no significant difference between URS and SWL (*I*^2^ = 91%, risk difference = 0.12, 95% CI − 0.10–0.33, *p* = 0.29) (Table 3 and Fig. 2c).

### Cost and hospital stay

**Fig. 3:**
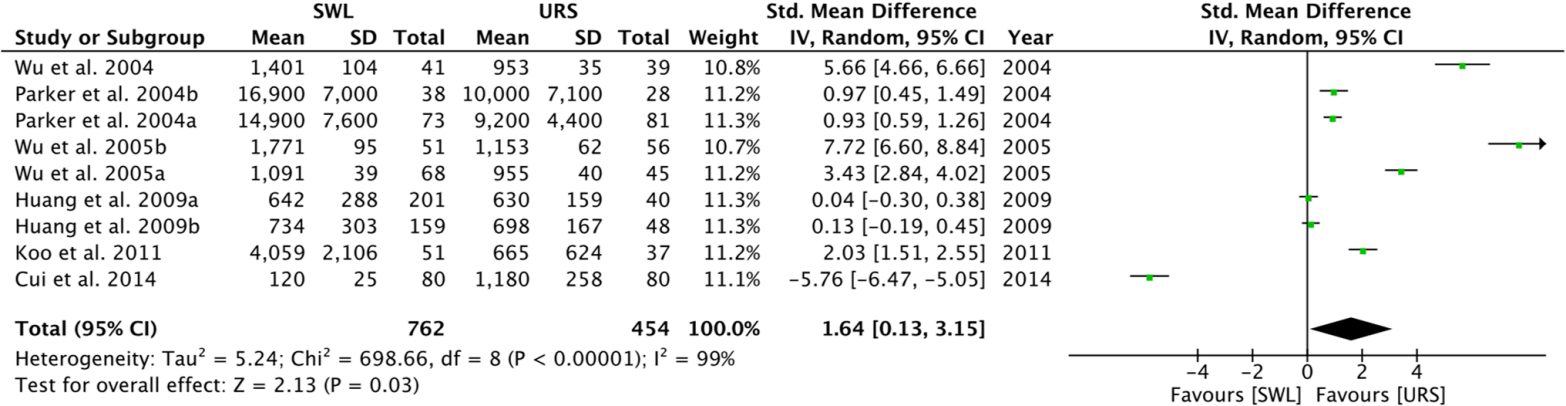
Forest plot of cost between SWL and URS

URS (mean: $2801) was significantly cheaper than SWL (mean: $3637) (standard mean difference = 1.64, 95% CI 0.13–3.15, *I*^2^ = 99%, *p* = 0.03) (Table 4 and Fig. 3). Cost breakdown is itemized in Table 5.

**Table 4:** SWL vs. URS cost data and hospital stay

Study	Price ($)		P (SWL vs. URS cost) from original studies	Hospital stay, days ± SD (range)	
	SWL ± SD	URS ± SD		SWL	URS
**Pearle et al. 2001** [11]	$7343	$6088	N/A	94% day-case	75% day-case
Parker et al. 2004 [12] < 10 mm	$14,900 ± 7600	$9200 ± 4400	<0.001	N/A	N/A
Parker et al. 2004 [12] ≥ 10 mm	$16,900 ± 7000	$10,000 ± 7100	<0.0001	N/A	N/A
Wu et al. 2004 [13]	$1401 ± 104	$953 ± 35	0.001	N/A	N/A
Wu et al. 2005 [14] < 10 mm	$1091.00 ± 39	$955 ± 40	0.01	N/A	N/A
Wu et al. 2005 [14] ≥10 mm	$1771 ± 95	$1153 ± 62	<0.001	N/A	N/A
Lee et al. 2006 [15]	$1637	$2154	N/A	1.8 ± 0.4	4.7 ± 2
Salem 2009 [16]	$1300	$1140	<0.05	N/A	N/A
Huang et al. 2009 [17] < 10 mm (overall)	$642 ± 288	$630 ± 159	0.47	2.0 ± 0.7	2.9 ± 1.4
Huang et al. 2009 [17] ≥ 10 mm (overall)	$734 ± 303	$698 ± 167	0.32	2.0 ± 0.7	2.9 ± 1.4
Huang et al. 2009 [17] < 10 mm (upper ureter)	$632 ± 114	$688 ± 212	0.04	2.0 ± 0.7	2.9 ± 1.4
Huang et al. 2009 [17] ≥ 10 mm (upper ureter)	$690 ± 130	$846 ± 232	0.03	2.0 ± 0.7	2.9 ± 1.4
Koo et al. 2011 [18]	$4059 ± 2106	$665 ± 624	<0.001	N/A	N/A
Cui et al. 2014 [19]	$120 ± 25	$1180 ± 258	<0.05	0.25 ± 0.7	2.8 ± 2.3
Cone et al. 2014 [20]	$3167	$4470	N/A	N/A	N/A
Cone et al. 2017 [21]	$3167	$4470	N/A	N/A	N/A
Chan et al. [22]	$931	$1564	<0.001	0 ± 0.4	2.4 ± 3.5
Total	$3637	$2801.33		1.2	3.1

*N/A* not available

**Table 5:** SWL vs. URS Itemized cost breakdown (as reported by individual studies)

Study	Cost breakdown and calculation for each study (based on the individual studies)		Study population
	SWL	URS	
Pearle et al. 2001 [11]	Hospital fee, anaesthesia professional fee, urology professional fee	Hospital fee, anaesthesia professional fee, urology professional fee, office stent removal, urology fee for stent removal	Adults with solitary radiopaque distal ureteric calculus below bony pelvis ≤ 15 mm
Parker et al. 2004 [12]	Initial procedure, additional procedures, radiographs, clinics	Initial procedure, additional procedures, radiographs, clinics	Adults with solitary radiopaque stone between ureteropelvic junction and sacroiliac joint
Wu et al. 2004 [13]	Pre-op evaluation, operation, perioperative monitoring, postoperative care, office visits, ancillary procedures and retreatment	Pre-op evaluation, operation, perioperative monitoring, postoperative care, office visits, ancillary procedures and retreatment	Adults with solitary upper ureteral (UPJ to SIJ) stone > 1 cm. Patient choice on treatment option
Wu et al. 2005 [14]	Pre-op evaluation, operation, perioperative monitoring, postoperative care, office visits and any ancillary/retreatment procedures	Pre-op evaluation, operation, perioperative monitoring, postoperative care, office visits, ancillary procedures and retreatment	Adults with single, primary, upper ureteral radiopaque calculus. Patient choice on treatment option
Lee et al. 2006 [15]	Hospital charges, operating room, radiology, surgeon, anaesthesia and auxiliary procedures	Hospital charges, operating room, radiology, surgeon, anaesthesia, auxiliary procedures and SWL machine	Adults with a solitary upper ureteral stone (above the border of L5 vertebra), ≥ 15 mm
Salem, 2009 [16]	Pre-op evaluation, operation, perioperative monitoring, postoperative care, office visits, ancillary procedures and retreatment	Pre-op evaluation, operation, perioperative monitoring, postoperative care, office visits, ancillary procedures and retreatment	Adults with single radiopaque upper ureteral stone 5–20 mm
Huang et al. 2009 [17]	Pre-op evaluation, operation, perioperative monitoring, postoperative care, office visits, ancillary procedures and retreatment	Pre-op evaluation, operation, perioperative monitoring, postoperative care, office visits, ancillary procedures and retreatment	Adults with ureteral stones (upper ureter defined as above). Unclear if solitary or lucency on XR
Koo et al. 2011 [18]	Procedural + overheads	Procedural + overheads	Adults with symptomatic radiopaque renal calculi < 20 mm
Cui et al. 2014 [19]	N/A	N/A	Adults with single radiopaque stone 8–15 mm. Patient choice on treatment
Cone et al. 2014 [20]	Surgeons fee, anaesthesia, facility cost, stent placement	Surgeons fee, anaesthesia, facility cost, stent placement	Adults with radiopaque renal stones < 15 mm. Patient choice on treatment
Cone et al. 2017 [21]	N/A	N/A	Adults with radiopaque ureteral stones < 15 mm. Patient choice on treatment
Chan et al. 2017 [22]	Cost per procedure (NHS tariff)	Cost per procedure (NHS tariff)	Adults with single radiopaque or radiolucent lower pole renal stones 10–20 mm

The mean hospital stay was significantly shorter for SWL (1.2 days, range: 0–2) compared to URS (3.1 days, range: 0–4.7).

### Sub-analyses

#### Stone size

Subanalysis of studies comparing SWL and URS was possible for stone size smaller than 10 or 10 mm and larger. Both groups favoured URS in terms of cost (< 10 mm: Std mean diff = 0.90, 95% CI 0.68–1.12, *I*^2^ = 98%, *p* < 0.001; ≥ 10 mm: Std mean diff = 0.78, 95% CI 0.51–1.04, *I*^2^ = 99%, *p* < 0.001).

#### Proximal ureteric stones

Proximal ureteric stones treated with URS had significantly cheaper costs (Std. mean diff = 0.99, 95% CI 0.82–1.15, *p* < 0.001).

#### Risk of bias analysis

**Fig. 4:**
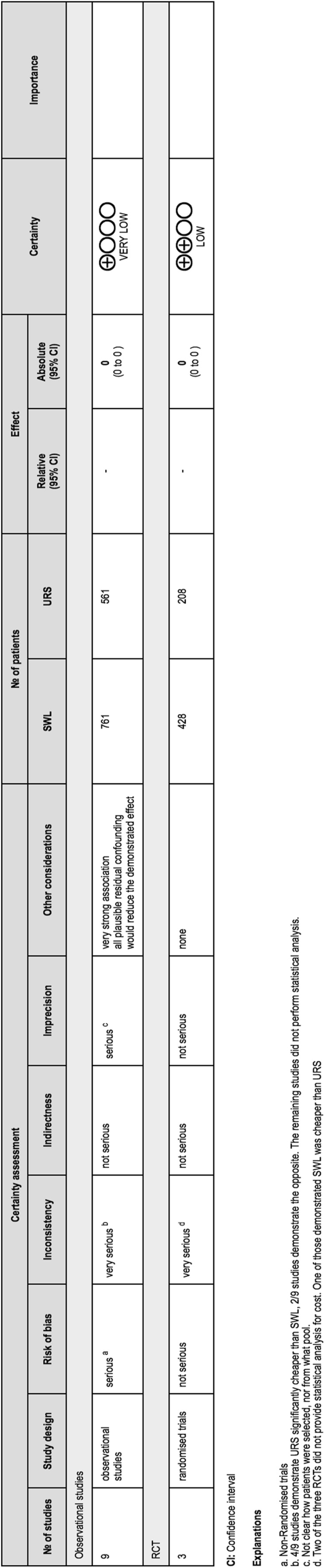
Risk of bias analysis

Risk of bias was analysed in each study (Fig. 4). The overall the risk of bias was high. There were only three prospective randomized trials with the remaining studies being prospective cohort studies (*n* = 2) and retrospective studies (*n* = 7). The randomized trials scored a ‘low’ certainty on bias analysis using GRADE, and the observational studies scored a ‘very low’ certainty.

None of the studies were blinded, although given that these were surgical studies blinding is not always feasible. The data was complete for all studies except these four studies [13, 15, 19, 20], however none of the studies provided a patient/study participant flow diagram to allow for easy assessment of attrition bias.

Reporting bias was suspected in two studies [19, 22]. Cui et al. [19]. performed a retrospective study with 80 patients in each treatment arm and it is possible that other patients were treated during the same time period but excluded from the study. Thus there is a high risk of selection and reporting bias. Chan et al. [22]. had very large numbers of SWL but relatively few patients undergoing URS, which reduced the power of the study increased the risk of Type II statistical error.

## Discussion

### Principal findings

This study is the first meta-analysis comparing cost of URS and SWL as reported by the authors and has comprehensively examined all objective outcome measures. The analysis shows that URS has higher SFR and lower retreatment rates in comparison to SWL. While the cost was significantly lower for URS, the complication rates were relatively higher than SWL although this was not statistically significant. URS seem to be more cost effective for treatment of stones of all sizes.

### Meaning of the study: implications for clinicians and policymakers

This review has demonstrated the overall advantage of URS in terms of cost, SFR and retreatment rates. While the calculation of costs across studies is not standardized, the comparison within each study is done using similar parameters. These costs might vary across various healthcare systems but considering that the studies have been reported from different countries, the results are generalizable to most patients. Treatment decisions should be individualized for patients after informed consent and should be based on clinical needs rather than economical compulsions.

### Strengths and limitations

The strength of our review is the systematic approach used to review the literature on the cost comparison for URS and SWL. Two independent researchers not involved in any of these reported studies performed the data extraction. Furthermore, a meta-analysis and risk of bias has also been conducted with sub-analysis of available stone parameters.

An obvious weakness of our review is the dependence on primary studies, which did not have standardized reporting of cost and comes from different healthcare setups where the treatment costs are variable. Similarly, the cost of URS has changed dramatically in the study period and new technologies adopted in the past few years have had a big influence on the cost.

The non-randomized studies were potentially prone to bias in patient selection and outcome reporting and the randomized studies were not blinded. Only Parker et al. [12] had a chance of blinding as their subjects underwent general anaesthesia (GA) for both URS and SWL, whereas all other studies used sedation for SWL rather than GA. While cost was calculated, their quality of life was not reported, which can be especially affected in patients undergoing URS with a post-operative stent insertion. None of the studies provided sample size/power calculations or CONSORT [23] flow diagrams for patient involvement in the study.

There was significant heterogeneity between the studies for all outcome measures. For continuous data like the cost and hospital stay, this was adjusted for by using standard mean difference and fixed effects analysis on forest plot. Heterogeneity would be expected given differences in available equipment, which could thereby affect outcomes and cost variation between countries. Cost also varied depending on what studies included in their ‘cost’, which would range from the procedure itself to the entire initial hospital stay plus office visits/additional hospital stays. Despite a lack of randomization, which is often unachievable in invasive surgical studies, [24] the outcome measures were objective and often dichotomous (i.e., SFR, retreatment rate, complication rate), therefore reducing the risk of a placebo effect.

### Strengths and weaknesses in relation to other studies, discussing important differences in results

Hospital stay was significantly longer for URS but given that only three studies provided this data and there was a high heterogeneity between the studies (*χ*^2^: *p* < 0.001, *I*^2^ = 80%), this result must be open to interpretation. Modern studies have demonstrated that day-case URS is becoming increasingly more feasible and therefore more comparable to SWL [25].

Decision analysis models were excluded as they do not include patient data. However, published decision analysis models can provide a useful tool to compare SWL and URS. Lotan et al. [26] demonstrated that above a SFR of 80% URS is more cost-effective than SWL. Another model by Cone et al. [20] demonstrated that a 67% SFR using SWL would be cost effective and a SFR < 71% using URS would not be cost effective, concluding that URS could be considered as a first-line treatment for renal or ureteric calculi < 1.5 cm in patients who desire to be stone free. Mean SFR for studies in this meta-analysis was 84%. Seven studies crossed this cost-effectiveness threshold, covering proximal ureteric stones and all renal stones [12, 13, 16, 17, 19–21]. The results also demonstrate a trend, reflected in another systematic review, that higher case volume results in higher SFR and fewer complications [27].

Although SWL is less invasive, over the last decade there is a shift from physician delivered to technician delivered treatment, perhaps coupled with a relative lack in technological advancements and investment in SWL when compared to URS. Optimization of SWL with training and proper maintenance can offer better treatment outcomes, which in turn can decrease the overall cost of SWL [28].

The limitations of our study relate to the heterogeneous nature of the studies included from different countries with variable practice patterns. The SFR was not defined consistently across studies [29]. Similarly the measurement of cost varied across studies although for each study as the cost of procedures would vary between healthcare systems and the author’s account of cost was taken into consideration.

### Future studies

There are large numbers of retrospective case series within the surgical literature suggesting ways to minimize costs [29, 30]. This constitutes a very poor evidence base on which to base recommendations. There needs to be a trend towards larger randomized trials that are powered towards the desired outcome, and therefore able to accurately assess the true cost-effectiveness. In addition to comparing the cost of SWL and URS it needs to include quality of life measurements, which is a significant cause of morbidity in especially in URS.

## Conclusion

There is limited evidence to suggest that URS is less expensive than SWL. However, due to lack of standardization, studies seem to be contradictory and further randomized studies are needed to address this issue.
